# The TOTUM-63 Supplement and High-Intensity Interval Training Combination Limits Weight Gain, Improves Glycemic Control, and Influences the Composition of Gut Mucosa-Associated Bacteria in Rats on a High Fat Diet

**DOI:** 10.3390/nu13051569

**Published:** 2021-05-07

**Authors:** Marine Dupuit, Vivien Chavanelle, Benoit Chassaing, Fanny Perriere, Monique Etienne, Claire Plissonneau, Audrey Boscaro, Nicolas Barnich, Vincent Pialoux, Thierry Maugard, Florian Le Joubioux, Sébastien Peltier, Pascal Sirvent, Yolanda F. Otero, Nathalie Boisseau

**Affiliations:** 1 Laboratoire des Adaptations Métaboliques à l’Exercice en conditions Physiologiques et Pathologiques (AME2P), Université Clermont Auvergne, CRNH Auvergne, 63000 Clermont-Ferrand, France; marine.dupuit@uca.fr (M.D.); monique.etienne@uca.fr (M.E.); claire.plissonneau@uca.fr (C.P.); audrey.boscaro@uca.fr (A.B.); 2 Valbiotis R&D, Riom Center, 63200 Riom, France; Vivien.Chavanelle@valbiotis.com (V.C.); pascal.sirvent@valbiotis.com (P.S.); yolanda.otero@valbiotis.com (Y.F.O.); 3 Inserm U1016, Team “Mucosal Microbiota in Chronic Inflammatory Diseases”, Université de Paris, CNRS UMR 8104, 75014 Paris, France; benoit.chassaing@inserm.fr; 4 Laboratoire Microorganismes: Génome et Environnement (LMGE), Université Clermont Auvergne, CNRS, 63000 Clermont-Ferrand, France; fanny.perriere@uca.fr; 5 Microbes, Intestin, Inflammation et Susceptibilité de l’Hôte (M2iSH), UMR 1071 Inserm, USC-INRAE 2018, Université Clermont Auvergne, CRNH Auvergne, 63000 Clermont-Ferrand, France; nicolas.barnich@uca.fr; 6 Laboratoire Interuniversitaire de la Biologie et de la Motricité (LIBM), Université Claude Bernard Lyon 1, EA 7424, 69266 Villeurbane, France; vincent.pialoux@univ-lyon1.fr; 7 UMR 7266 CNRS-ULR, LIENSs, Equipe BCBS, La Rochelle Université, 17042 La Rochelle, France; thierry.maugard@univ-lr.fr; 8 Valbiotis R&D, La Rochelle Center, 17000 La Rochelle, France; florian.lejoubioux@valbiotis.com (F.L.J.); sebastien.peltier@valbiotis.com (S.P.)

**Keywords:** high-intensity interval training, plant extract supplementation, glycemic control, body composition, microbiota

## Abstract

Obesity and prediabetes are the two strongest risk factors of type 2 diabetes. It has been reported that TOTUM-63, a polyphenol-rich plant extract, has beneficial effects on body weight (BW) and insulin resistance in mice fed a high fat diet (HFD). The study aim was to determine whether high-intensity interval training (HIIT) and/or TOTUM-63 supplementation improved body composition and glycemic control and gut microbiota composition in a Western diet-induced obesity rat model. Wistar rats received a standard diet (CTRL; control; *n* = 12) or HFD (HFD; *n* = 48) for 16 weeks. Then, HFD rats were divided in four groups: HFD, HFD + TOTUM-63 (T63), HFD + HIIT (HIIT), and HFD + HIIT +T63 (HIIT + T63). Training was performed 4 days/week for 12 weeks. TOTUM-63 was included in diet composition (2%). The HIIT + T63 combination significantly limited BW gain, without any energy intake modulation, and improved glycemic control. BW variation was correlated with increased α-diversity of the colon mucosa microbiota in the HIIT + T63 group. Moreover, the relative abundance of *Anaeroplasma, Christensenellaceae* and *Oscillospira* was higher in the HIIT + T63 group. Altogether, these results suggest that the HIIT and TOTUM-63 combination could be proposed for the management of obesity and prediabetes.

## 1. Introduction

Type 2 Diabetes (T2D) prevalence has been increasing worldwide in the last decades [1]. Overweight/obesity and prediabetes (a non-pathological stage characterized by increased fasting blood glucose, postprandial glucose and/or elevated hemoglobin A1c [2]) are the two strongest risk factors of T2D development [3]. Indeed, 5–10% of patients with prediabetes typically progress to T2D each year [4], and 70% will develop T2D during their lifetime [5]. Obesity, which is characterized by excessive accumulation of adipose tissue and large (intra-)abdominal fat mass (FM), is correlated with insulin resistance [6], and increases by 95% the risk of T2D [7]. Thus, preventing and treating prediabetes and obesity are relevant strategies for avoiding or slowing down T2D.

Lifestyle interventions, including diet and/or physical activity programs, may help in this context. The American College of Sports Medicine has recommended low- to moderate-intensity continuous training for patients with obesity and (pre)diabetes [8]. Currently, high-intensity interval training (HIIT), which includes repeated bouts of high-intensity effort followed by varied recovery times [9], is considered a time-efficient and safe strategy to reduce total FM, particularly intra-abdominal FM [10,11]. Moreover, many studies have reported glucose homeostasis improvement following HIIT programs [12,13,14]. HIIT effects on glycemic control could be partly explained by an increase in the skeletal muscle oxidative capacity and higher glucose transport activity [15]. In addition, a growing body of evidence suggests that combining HIIT and nutritional strategies (such as plant extract supplementations) may result in more favorable outcomes by enhancing energy metabolism, or by increasing the adaptive response during recovery [16].

TOTUM-63 (T63) is a polyphenol-rich extract obtained from five plants (olive leaves, bilberry, artichoke, chrysanthellum, and black pepper) and designed to reduce T2D risk factors. T63 has positive effects on body composition and whole-body glucose homeostasis in high fat diet (HFD)-fed mice [17]. In these mice, addition of T63 in the food for 16 weeks prevents diet-induced excessive weight and FM gains and limits the HFD effects on glucose homeostasis. Specifically, fasting insulin levels were reduced, the insulin response to an oral glucose challenge was improved, and insulin-stimulated AKT phosphorylation in skeletal muscle and adipose tissue was restored [17]. In humans, T63 shows good tolerability and safety, and T63 supplementation for 4 weeks (5g·d^−1^) improves the response to an oral carbohydrate tolerance test. This suggests that T63 is a promising candidate for glucose management.

T63 also shows beneficial effects on gut microbiota, by partially restoring the α and β diversity index and by improving bacterial taxonomy in mice [17]. Gut microbiota dysbiosis has been proposed as a possible contributor to obesity and metabolic diseases, such as T2D [18]. Therefore, improving intestinal microbiota could be an innovative therapeutic target for diabetes prevention [19]. Gut microbiota composition and function are influenced by regular physical activity and diet interventions [20]. Furthermore, several authors suggest that physical activity effects on gut microbiota composition and function could improve glycemic control in subjects with (pre)-diabetes [19]. Physical activity could restore gut microbiota equilibrium by enhancing the number of beneficial bacteria and simultaneously reducing pathogenic bacteria and by improving microbiota diversity [21].

On the basis of these data, the aim of this study was to evaluate the effects of a 12-week intervention program that included physical activity (HIIT) and/or T63 supplementation on body composition and glycemic control in a rodent model of obesity. We first hypothesized that HIIT and T63 would positively affect whole body FM and whole body glucose homeostasis, and that their combination (HIIT + T63) might induce greater positive effects. We also hypothesized that each intervention could specifically affect the mucosa-associated gut microbiota composition (α and β diversity), with more favorable adaptations in the HIIT + T63 group. A better understanding of the effects of physical activity, T63 consumption, and their potential interaction will help to develop personalized intervention strategies to reduce adiposity and glucose homeostasis impairment and ultimately T2D development.

## 2. Materials and Methods

### 2.1. Animals Characteristics

The required sample size (*n* = 8 animals/group) was calculated on the basis of the main outcome (glycemic control) with a statistical significance of <0.05, power of 0.8, and assuming a variance of 25% in the outcome of interest. Additional animals were added in each group to account for the refusal to exercise by some rats (about 10–20%). This led to a total of 60 male Wistar rats (Charles River, France). Rats (8 weeks of age) were housed in individual cages in controlled conditions (22 ± 1 °C; 12-h light/12-h dark cycle). All experiments were approved by the local ethics committee (C2EA-02, Auvergne, France; APAFIS 15853-2018070413182792), and were performed in accordance with the animal welfare regulations and guidelines (European Directive 2010/63/EU on the protection of vertebrate animals used for experimental and scientific purposes). All efforts were made to protect animal welfare and to minimize suffering at each protocol step.

### 2.2. Intervention

**Figure 1:**
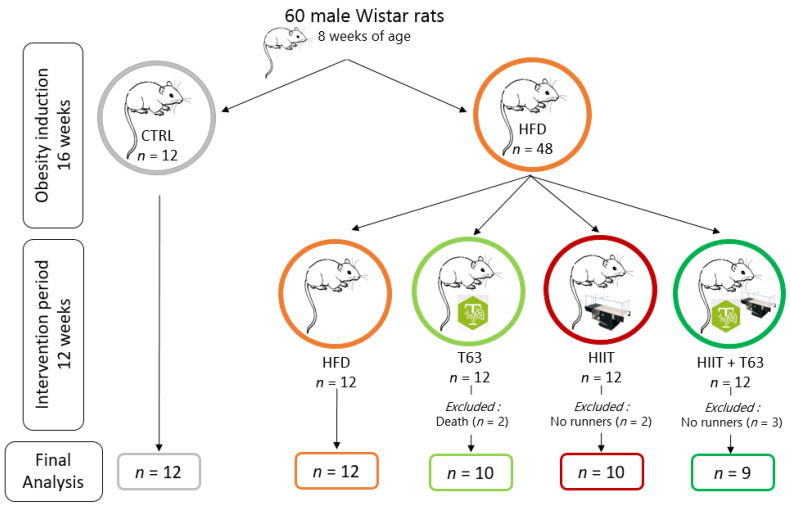
Study design. Rats were randomized in two groups: control (CTRL; *n* = 12) and High-Fat Diet (HFD; *n* = 48) for 16 weeks (obesity induction). Then, the HFD group was divided in four groups (*n* = 12/group) matched for weight, fat mass and fasting blood glucose: HFD, TOTUM-63 (T63), High-Intensity Interval Training (HIIT), and HIIT + T63 (intervention period for 12 weeks). In the HIIT and HIIT + T63 groups, “no runner” rats were excluded from the study. Two rats from the T63 group died during glucose gavage due to intubation mishandling. Finally, 41 rats were included in the analysis (HFD: *n* = 12; T63: *n* = 10; HIIT: *n* = 10 and HIIT + T63: *n* = 9).

After one week of acclimation, animals were distributed in two dietary groups (food ad libitum): high-fat diet (HFD, *n* = 48; 45% of kcal from fat) (#D12451, Research Diets, Inc., New Brunswick, NJ, USA), and standard diet (CTRL, *n* = 12; 10% of kcal from fat) (#D12450H, Research Diets, Inc., New Brunswick, NJ, USA). After 16 weeks of obesity induction, animals in the HFD group were divided in four groups matched for body weight (BW), fat mass (FM) and fasting glycemia: (i) HFD alone (HFD group), (ii) HFD + HIIT (HIIT group), (iii) HFD + T63 supplementation (T63 group), and (iv) HFD + HIIT + T63 (HIIT + T63 group). T63 was added to the HFD at a dose of 2% (#D18091303, Research Diets, Inc., New Brunswick, NJ, USA) The T63 supplement contains extracts from olive leaves (*Olea europaea*), bilberry (*Vaccinium myrtillus*), artichoke (*Cynara scolymus*), chrysanthellum (*Chrysanthellum indicum subsp. afroamericanum* B.L. Turner) and black pepper (*Piper nigrum*). Table 1 summarizes T63 chemical characterization with the estimated total polyphenols and total fibers and the quantification of all other potential compounds of interest. The intervention was implemented for 12 weeks, from week 16 to week 28 (Figure 1).

Rats were euthanized after isoflurane anesthesia by exsanguination through cardiac puncture. Blood, skeletal muscle (i.e., gastrocnemius and soleus), white adipose tissues (i.e., epididymal, mesenteric and subcutaneous), brown adipose tissue, colon, cecal content, and liver were collected and stored appropriately for later analysis. Samples were collected at least 72 h after the last exercise session to avoid any interference of physical exercise acute effects on the analyses.

### 2.3. Food Consumption, Body Weight and Body Composition

Food consumption (g) and BW (g) were assessed three times per week. Food consumption corresponded the difference between the weight of the food given and the weight of the unconsumed food. This value and the energy values of each diet were used to calculate the energy intake (kCal). FM and lean body mass (LBM) were measured with an EchoMRI device (EchoMRI Medical System, Houston, TX, USA) every four weeks.

### 2.4. Training Protocol

Rats in the HIIT (*n* = 12) and HIIT + T63 (*n* = 12) groups underwent a 10-day acclimation on a treadmill (Matsport, France) during which speed and running time were progressively increased. Then, rats performed the 12-week HIIT program, 4 days per week, as follows: 6 × (3 min at 10 m·min^−1^/4 min at 18 m·min^−1^), for a total of 42 min/training session.

### 2.5. Fasting Glycemia, Fasting Insulinemia, and Oral Glucose Tolerance Test

Oral glucose tolerance tests (OGTT) were performed at week 1, 16 and 28. Briefly, after 6h of fasting, the baseline glucose level (t = 0 min) was measured in tail vein blood (approximately 2 μL) using a standard glucose meter (Accu Check Performa^®^, Roche Diabetes Care, Montbonnot-Saint-Martin, France) to determine the fasting glycemia. Then, rats received glucose by gavage (2 g·kg^−1^ of lean body mass), and glucose concentration was measured in blood samples collected from the tail vein at 30, 60, 90, and 120 min after the oral glucose load. The total area under the curve (AUC) was calculated using the trapezoidal method. The plasma concentration of insulin was measured with a commercial ELISA kit (Alpco, Salem, NH, USA) in tail blood samples at 0 (i.e., fasting insulinemia), 30 and 120 min after the oral glucose load. The Insulin-Resistance index (HOMA-IR) was determined with the following formula: HOMA-IR = (fasting insulinemia (μU·mL^−1^) × fasting glycemia (mM))/22.5 [22].

### 2.6. Citrate Synthase Activity

Citrate synthase activity, a marker of muscle oxidative activity [23], was measured in soleus and gastrocnemius muscle homogenates according to the method described by Stephenson et al. [24].

### 2.7. Protein Extraction and Western Blotting

Protein lysates from frozen soleus, gastrocnemius and colon were prepared using RIPA buffer (50 mM Tris-HCl, 150 mM NaCl, 1% Nonidet P-40, 0.50% sodium deoxycholate, 0.10% sodium dodecyl sulfate, pH: 8.0) supplemented with freshly added protease inhibitor cocktail (P8340, Sigma Aldrich, Saint-Louis, MO, USA) and phosphatase inhibitor tablets (#88667 Thermo Fisher Scientific, Whaltham, MA, USA). Tissues were homogenized on ice using a glass Potter homogenizer followed by 1h incubation at 4 °C. Samples were centrifuged at 14,000× *g* for 10 min (+4 °C), and supernatants were collected. The supernatant protein content was determined using the commercial DC protein assay (Bio-Rad, USA). Western blotting was performed as described by Chavanelle et al. [25] with the following modifications. Membranes were incubated overnight at 4 °C with primary antibodies against glucose transporter type 4 (GLUT4) (IF8) (1:1000; Cell signaling, Danvers, MA, USA), and OXPHOS complexes (1:4000; Abcam, Cambridge, MA, USA). After incubation, membranes were washed three times with tris-buffered saline/0.1% Tween 20 (TTBS) and incubated with the appropriate dilutions of anti-species horseradish peroxidase-conjugated secondary antibodies at room temperature for 1 h. Then, membranes were washed three times in TTBS before addition of the enhanced chemiluminescent solution (Clarity Western ECL; Bio-Rad, Hercules, CA, USA) for 5 min, using the Bio-Rad ChemiDoc system. Band densities were determined using Image Lab V5.0 (Bio-Rad, USA). The Stain Free^®^ (Bio-Rad, USA) total protein measurement was used as loading control and for data normalization [26].

### 2.8. RNA Isolation, Reverse Transcription and Quantitative Real-Time PCR

Total RNA was extracted from muscles (30 mg), colon (50–60 mg), and adipose tissue (50–60 mg) using TRIzol^®^ (Invitrogen, Life Technologies, Carlsbad, CA, USA). In adipose tissue, total RNA was purified with the NucleoSpin^®^ RNA Set (Marcherey-Nagel, Hoerdt, France). cDNA was synthesized from 2 μg RNA with the High Capacity cDNA Reverse Transcription Kit (Applied Biosystems, Life Technologies). The relative expression of *Il1β*, *IL6*, *IL10*, *Tnfα*, *Ppargc1α* and *Nrf1* were evaluated by real-time PCR amplification using the CFX Bio-Rad system with SYBR^®^Green probe sets (Applied Biosystems, Beverly, MA, USA) (primer sequences in Table 2). Results were normalized to the expression level of glyceraldehyde 3-phosphate dehydrogenase (*Gapdh*), and gene expression was quantified with the ΔΔCt method. Data are presented using the Rq normalized to the control group, or Rq = 2^−ΔΔCt^ (ΔCt = Ct (target) − Ct (Gapdh); ΔΔCt = ΔCt (sample) − ΔCt (control)).

### 2.9. Other Biochemical Analyses

Advanced oxidation protein products (AOPP) were determined in plasma, liver, gastrocnemius and epididymal adipose tissue samples, as described by [27]. Oxidized Low-Density Lipoproteins (oxLDL) were measured in plasma using an ELISA kit (Elabscience^®^, Houston, TX, USA) according to the manufacturers’ recommendations. SuperOxide Dismutase (SOD), Glutathione Peroxidase (GPx) and catalase activities were determined in plasma, liver, muscle and epididymal adipose tissue, as described by Groussard et al. [27].

Plasma myeloperoxidase (MPO) concentration was measured using a commercial ELISA kit (Myeloperoxidase DuoSet ELISA, R&D Systems, Minneapolis, MN, USA).

Plasma lipopolysaccharide binding protein (LBP) was quantified with the LBP ELISA Kit for various species (Hycult Biotech, Frontstraat, Uden, The Netherlands), following the manufacturer’s instructions.

### 2.10. Concentration of Fecal Short Chain Fatty Acids (SCFAs) by Gas Chromatography

Weighed fecal samples were ground with Milli-Q^®^ water (500 µL for 100 mg), incubated at 4 °C for 2 h, and centrifuged at 12,000× *g* at 4 °C for 15 min. Supernatants were weighted, and saturated phosphotungstic acid solution was added (1 g for 100 μL). After overnight incubation at 4 °C, samples were centrifuged again, and SCFAs (acetate, butyrate and propionate) concentration were determined by gas chromatography (Agilent Technologies™ 6850, Santa Clara, CA, USA) using DB-FFAP columns (30 m × 250 μm, 0.25 μm). The temperature of the inlet was 250 °C and the injection volume was 1 μL. The temperature program was as follows: initial oven temperature: 100 °C, ramped to 250 °C (10 °C·min^−1^), and held for 5 min. The carrier gas was helium at a constant flow of 0.7 mL·min^−1^. Samples were imported into the column using the split mode at a ratio of 10:1. Detection was performed with the flame ionization detector.

### 2.11. Microbiota Composition Analysis by Illumina Sequencing

Genomic DNA from colon samples was lysed in proteinase K at 56 °C in a shaking incubator overnight. DNA was extracted using the Nucleospin^®^ Tissue Kit (Macherey-Nagel, Hoerdt, France). DNA concentration was determined with a QubitTM Fluorometer (Invitrogen), and DNA quality (260/280 and 260/230 ratios) was assessed with a NanoDrop spectrophotometer (ThermoScientific, Courtaboeuf, France). The *16S* rRNA gene was amplified and sequenced using the Illumina MiSeq technology and the Earth Microbiome Project protocol with some slight modifications in collaboration with the ‘‘Mucosal microbiota in chronic inflammatory diseases’’ research group (Inserm U1016, CNRS UMR 8104, Université de Paris, France). Briefly, region V4 of the *16S* rRNA gene was PCR-amplified from each sample using composite forward and reverse primers designed with the Golay error-correcting scheme, and used to tag the PCR products [28]. The sequence of the forward primer (515F) was: 5′-*AATGATACGGCGACCACCGAGATCTACACGCT*XXXXXXXXXXXX**TATGGTAATT*GT***GTGYCAGCMGCCGCGGTAA-3′. The italicized sequence is the 5′ Illumina adapter, the 12 X sequence is the Golay barcode, the bold sequence is the primer pad, the italicized and bold sequence is the primer linker, and the underlined sequence is the conserved bacterial primer 515F. The sequence of the reverse primer (806R) was: 5′-*CAAGCAGAAGACGGCATACGAGAT***AGTCAGCCAG*CC*** GGACTACNVGGGTWTCTAAT-3′. The italicized sequence is the 3′ reverse complement sequence of the Illumina adapter, the bold sequence is the primer pad, the italicized and bold sequence is the primer linker, and the underlined sequence is the conserved bacterial primer 806R. PCR reactions included the Hot Master PCR mix (Quantabio, Beverly, MA, USA), 0.2 mM of each primer, and 10–100 ng template. The reaction conditions were 3 min at 95 °C, followed by 30 cycles of 45 s at 95 °C, 60 s at 50 °C, and 90 s at 72 °C on a BioRad thermocycler. PCR products were quantified with the Quant-iT PicoGreen dsDNA assay. Then, a master DNA pool was generated from the purified products in equimolar ratios and purified with Ampure magnetic purification beads (Agencourt, Brea, CA, USA). The pooled product was quantified using the Quant-iT PicoGreen dsDNA assay and then sequenced using an Illumina MiSeq sequencer (paired-end reads, 2 × 250 bp) at Cornell University, Ithaca.

Using Quantitative Insights Into Microbial Ecology (QIIME2, Flagstaff, AZ, USA) version 2019.7, *16S* rRNA sequences were analyzed [29]. Sequences were demultiplexed and quality-filtered using the Dada2 method [30] with QIIME2 default parameters to detect and correct Illumina amplicon sequence data, and a table of QIIME2 artifacts was generated. Then, the α-diversity of bacterial communities was assessed by calculating the Shannon’s diversity index based on the number of observed operational taxonomic units (OTU). β-diversity was used to analyze the dissimilarity among the group membership and structure. Therefore, at 9103 sequencing depth, based weighted/unweighted UniFrac distances were reported according to the principal coordinate analysis (PCoA). Group differences in α and β-diversity indices were calculated using the Kruskal–Wallis test and permutational multivariate analysis of variance (PERMANOVA), respectively. For taxonomy analysis, features were assigned to OTUs with a 99% threshold of pairwise identity to the Greengenes reference database 13.8 [31]. Differential taxon abundance among groups was tested with ANCOM [32]. The W-value generated by ANCOM is a count of the number of sub-hypotheses (Aitchison’s log-ratio) that are significantly different across the tested groups for a given taxon. Correlations between gut microbiota composition (the relative abundance of features) and body or glycemic parameters were calculated with the Spearman’s correlation and the GraphPad Prism software (version 7.0).

### 2.12. Statistical Analysis

Statistical analyses were performed using the Statistica software (version 12.0). All data are presented as mean ± standard deviation (SD). Normality was verified using the Kolmogorov-Smirnov’s test, and homogeneity of variance was assessed with the Bartlett F-test. In the absence of normal distribution or variance homoscedasticity, data were log-transformed before analysis. One-way-ANOVA with the Newman Keuls’s post-hoc test was used to compare the CTRL and HFD groups (effects of obesity induction). Then, one-way ANOVA (with or without repeated measures) followed by the Newman-Keuls post-hoc test was used to determine group effects, and time (T), group (G) and T × G interactions in the four experimental groups (HFD, T63, HIIT and HIIT + T63). Spearman correlations were used to test relationships between variables. Differences were considered significant when *p*-values < 0.05. For easy comparison, the CTRL group values during the intervention period are presented with a gray dotted line/bar in the figures.

## 3. Results

### 3.1. HFD for 16 Weeks Significantly Alters Body Composition in Wistar Rats

**Figure 2:**
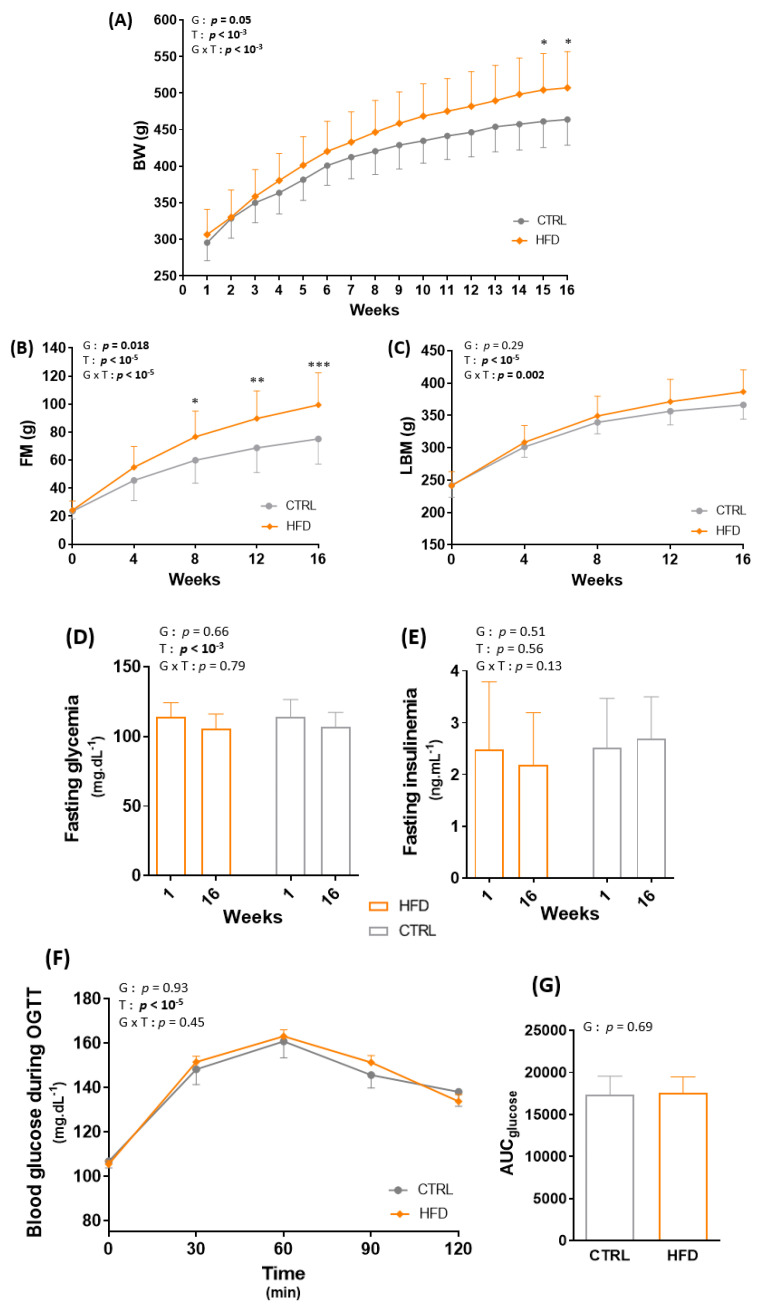
Effect of obesity induction on body weight (BW) (**A**), total fat mass (FM) (**B**), total lean body mass (LBM) (**C**), fasting glycemia (**D**), fasting insulinemia (**E**), blood glucose during the oral glucose tolerance test (OGTT) (**F**) and total area under the curve of glucose (AUC_glucose_) (**G**). * *p* < 0.05, ** *p* < 0.005, *** *p* < 0.0005, G: group effect, T: time effect, G × T: group × time effect. CTRL: control group, HFD: high-fat diet.

After 16 weeks of ad libitum HFD, BW and total FM (g and %) were significantly increased in the HFD compared with the CTRL group (*p* < 0.05, Figure 2A,B) without difference in LBM (*p* = 0.29) (Figure 2C). Despite the significant body composition changes, fasting glycemia and insulinemia and glucose tolerance were similar in the HFD and CTRL groups (Figure 2D–G).

After the 16-week induction period, animals in the HFD group were divided in four homogeneous groups (*n* = 12) matched for BW and FM (Figure 1). The 12 rats in the HFD group became the control group to assess the effect of training and/or T63 supplementation during the next 12 weeks.

### 3.2. The T63-HIIT Combination Limits Body Weight and Fat Mass Gain, Independently of the Energy Intake

**Figure 3:**
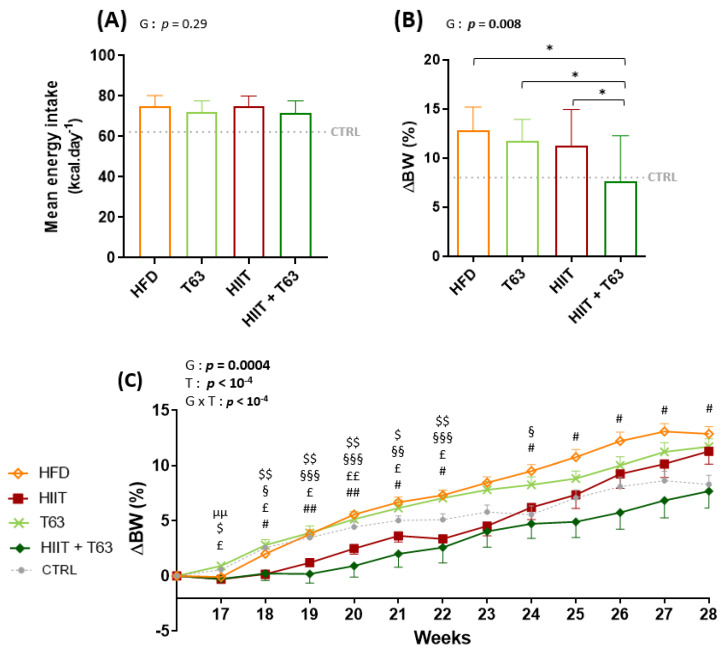

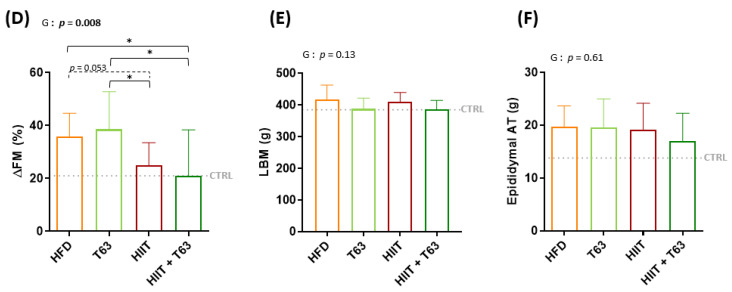
Mean energy intake in the four groups (**A**). Changes in body weight (ΔBW) (**B**,**C**), fat mass (ΔFM) (**D**), lean body mass (LBM) (**E**), and epididymal adipose tissue (AT) (**F**) at the end of the 12-week intervention. * *p* < 0.05, ^µµ^ *p* < 0.005: HFD vs. T63, ^$^ *p* < 0.05, ^$$^ *p* < 0.005: HIIT vs. T63, ^§^ *p* < 0.05, ^§§^ *p* < 0.005, ^§§§^ *p* < 0.0005: HIIT vs. HFD, ^£^ *p* < 0.05, ^££^ *p* < 0.005: HIIT + T63 vs. T63, ^#^ *p* < 0.05, ^##^ *p* < 0.005: HIIT + T63 vs. HFD. ΔBW = ((Week 28 BW − Week 16 BW)/Week 16 BW) × 100; ΔFM = ((Week 28 FM − Week 16 FM)/Week 16 FM) × 100, G: group effect, T: time effect, G × T: group × time effect.

The mean energy intake during the 12-week intervention was not different in the four groups (Figure 3A). At the end of the intervention, BW change (%) was lower in the HIIT + T63 group (Figure 3B). Interestingly, BW change (%) was comparable in the HIIT and HIIT + T63 groups until week 24, but only the HIIT and T63 association allowed limiting BW gain in the last four weeks (Figure 3C). At the end of the intervention, FM gain (% of change) was lower in the HIIT groups (HIIT + T63 vs. HFD and T63 groups: *p* = 0.028 and *p* = 0.016, respectively; HIIT vs. T63: *p* = 0.046; HIIT vs. HFD: *p* = 0.053) (Figure 3D). Conversely, LBM and epididymal AT were comparable in the four groups (Figure 3E,F).

### 3.3. The HIIT-T63 Combination Improves Glycemic Control

**Figure 4:**
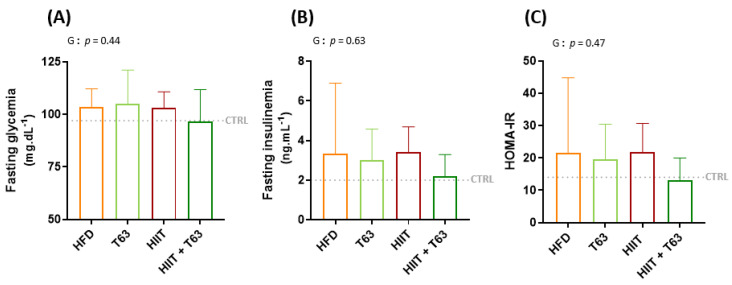

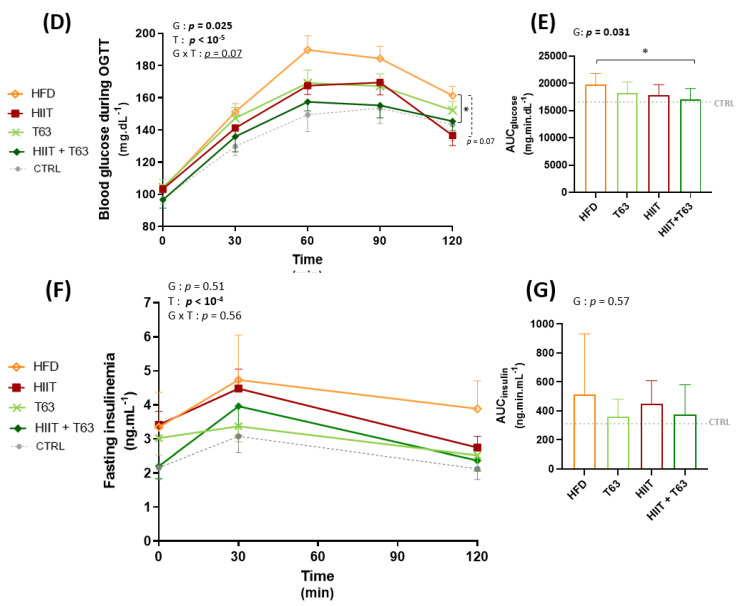
Effects of T63 supplementation and/or HIIT on (i) Fasting glycemia (**A**), Fasting insulinemia (**B**), and HOMA-IR (**C**) and on (ii) blood glucose (**D**,**E**), and insulin (**F**,**G**) levels during the oral glucose tolerance test (OGTT). * *p* < 0.05. AUC: area under the curve, G: group effect, T: time effect, G × T: group × time effect.

After 12 weeks of intervention (supplementation and/or physical training), fasting glycemia and insulinemia were comparable among groups, leading to similar HOMA-IR values (Figure 4). A significant group effect was observed for the blood glucose values during OGTT between the HIIT + T63 and HFD groups (*p* = 0.025, Figure 4D). Similarly, the AUC_glucose_ value was lower in the HIIT + T63 than in the HFD group, indicating that the T63 and HIIT combination improved glycemic control (Figure 4E). A trend was only observed when comparing the HIIT and HFD groups (*p* = 0.07). The insulin response was not different among groups (Figure 4F,G).

### 3.4. The HIIT-T63 Combination Improves Muscle Oxidative Metabolism

**Figure 5:**
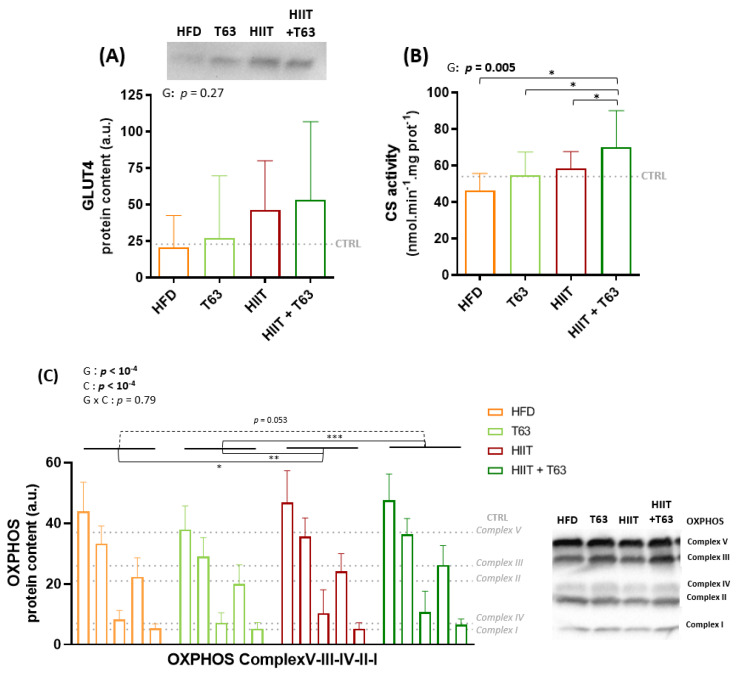
Effects of T63 supplementation and/or HIIT on GLUT4 protein content (**A**), citrate synthase (CS) activity (**B**), and OXPHOS complex protein content (**C**) in soleus. * *p* < 0.05, ** *p* < 0.005, *** *p* < 0.0005, G: group effect, T: time effect, G × T: group × time effect.

The GLUT4 protein level in the soleus muscle was not different among groups, although it tended to be higher in rats undergoing training (Figure 5A). Citrate synthase activity was significantly higher in the HIIT + T63 than in all the other groups (*p* < 0.05) (Figure 5B). Western blot analysis showed a significant global increase in the total amount of OXPHOS complex proteins (Figure 5C) in the two HIIT groups vs. the T63 group (group effect: *p* < 0.05), and in the HIIT vs. HFD group (*p* = 0.025). The difference was not significant between the HIIT + T63 and HFD groups (*p* = 0.053). No difference among groups was observed in gastrocnemius samples (Supplementary Figure S1).

No change was observed in the expression of *Ppargc1α* and *Nrf1,* two genes encoding regulators of mitochondrial biogenesis (Supplementary Figure S2).

### 3.5. T63 Supplementation and/or HIIT Enhances the Pro-/Anti-Oxidant Balance in Plasma

**Figure 6:**
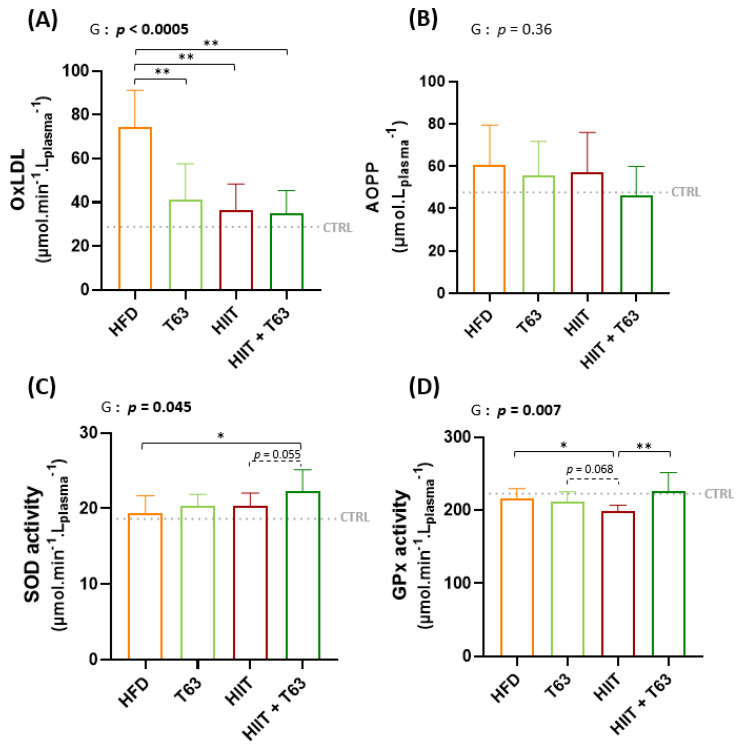
Effects of T63 supplementation and/or HIIT on oxidized low-density lipoproteins (oxLDL) (**A**), advanced oxidation protein products (AOPP) (**B**), superoxide dismutase (SOD) activity (**C**), and glutathione peroxidase (GP_X_) activity (**D**) in plasma. * *p* < 0.05, ** *p* < 0.005, G: group effect.

T63 supplementation and/or HIIT decreased HFD-induced oxidative stress, as shown by the lower oxLDL values (Figure 6A). AOPP concentration was not different among groups, although values were lower in the HIIT + T63 group (Figure 6B). Plasma SOD activity was higher only in the HIIT + T63 group (*p* = 0.025 vs. HFD) (Figure 6C), and plasma GP_X_ activity was higher in the HIIT + 63 and HFD groups compared with the HIIT group (*p* = 0.003 and *p* = 0.042, respectively) (Figure 6D). No change was observed in the other tissues examined (gastrocnemius, adipose tissue and liver) (Supplementary Figure S3).

In plasma, MPO and LBP values were not different among groups (Supplementary Figure S4). Expression of the *Il10*, *Il1β* and *Il6* genes in colon, epididymal adipose tissue, and subcutaneous adipose tissue was comparable among groups. *Il10* was not detected in subcutaneous adipose tissue and its expression levels in colon and epididymal adipose tissue were similar among groups (Supplementary Figure S5)

### 3.6. T63 and HIIT Differently Modulate the Intestinal Mucosa-Associated Microbiota

**Figure 7:**
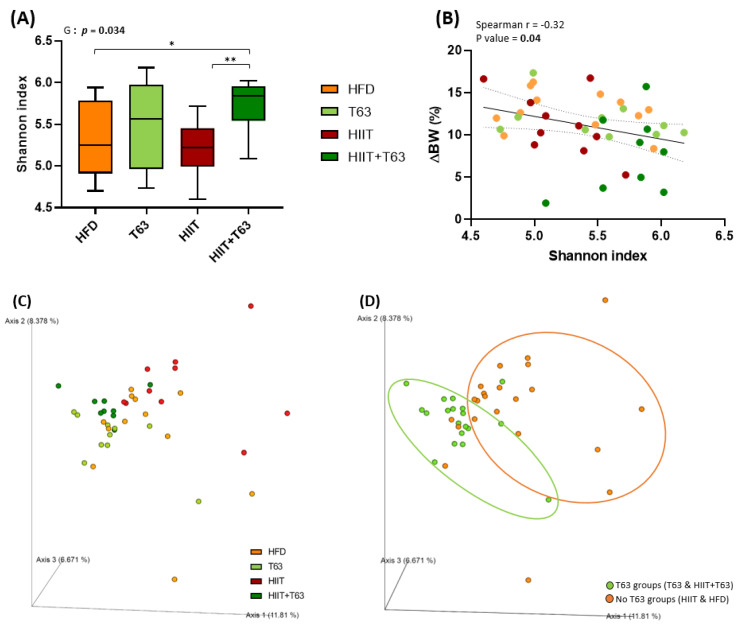
Mucosa-associated microbiota composition analyzed by *16S* rRNA gene sequencing in colon DNA samples (Illumina MiSeq system). Shannon index (**A**), correlation between Shannon index and body weight change (%) (ΔBW) (**B**). Unweighted Unifrac analysis clustered in four groups (**C**) and two groups (T63 and no T63) (**D**), G: group effect. * *p* < 0.05, ** *p* < 0.005.

Analysis of the composition of the colonic mucosa microbiota by *16S* rRNA sequencing revealed that the α-diversity (Shannon diversity index) was different among groups (*p* = 0.034), and was significantly increased in the HIIT + T63 group (*p* = 0.023 and *p* = 0.002 *vs* the HFD and HIIT groups, respectively) (Figure 7A). Interestingly, the Shannon index was negatively correlated with BW variations (∆BW) (Figure 7B), suggesting an association between rich microbiota and protection against metabolic deregulation.

β-diversity analysis through computation by PCoA of unweighted Unifrac distance matrices highlighted two main clusters based on the presence/absence of T63 supplementation (Figure 7C,D). Indeed, although β-diversity was different between the HIIT and HFD groups, T63 displayed the main effect on the composition of the mucosa-associated microbiota (*p* = 0.001 for T63 vs. HIIT; *p* = 0.001 for HIIT + T63 vs. HIIT and HFD), demonstrating the ability of the T63 extract to modulate the mucosa-associated colonic microbiota.

The HIIT and T63 combination may differently influence metabolites or specific features compared with HIIT and T63 alone. As SCFAs are derived from the microbiota, major SCFAs (acetate, butyrate and propionate) were quantified in fecal samples to evaluate the microbiota global fermentative-metabolic capacity. Their concentrations and the total SCFA amount (acetate + butyrate + propionate) were similar among groups after the 12-week intervention (Table 3).

**Figure 8:**
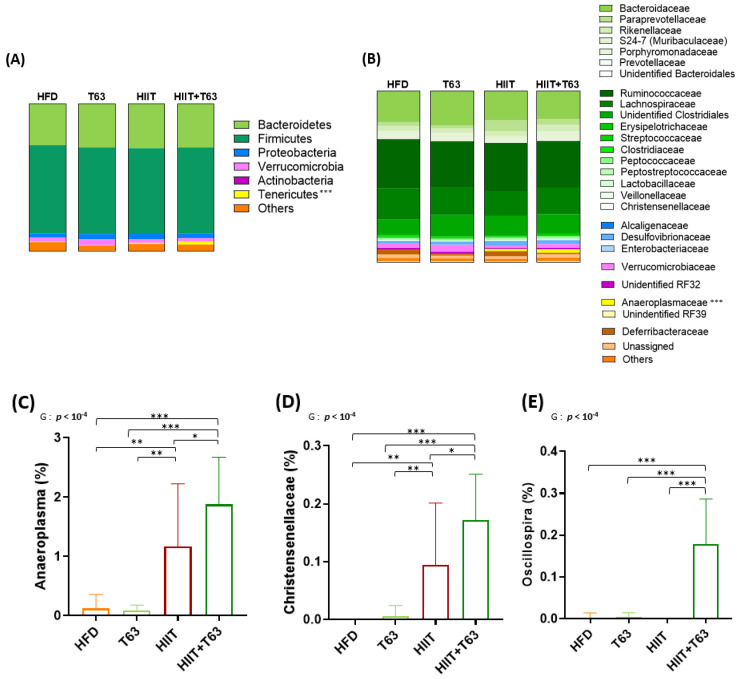
Taxonomy at the phylum (**A**) and family (**B**) levels for the four groups. Relative abundance (%) of specific bacterial types in the mucosa-associated microbiota at the end of the 12-week intervention (**C**–**E**) * *p* < 0.05, ** *p* < 0.005, *** *p* < 0.0005, G: group effect.

**Figure 9:**
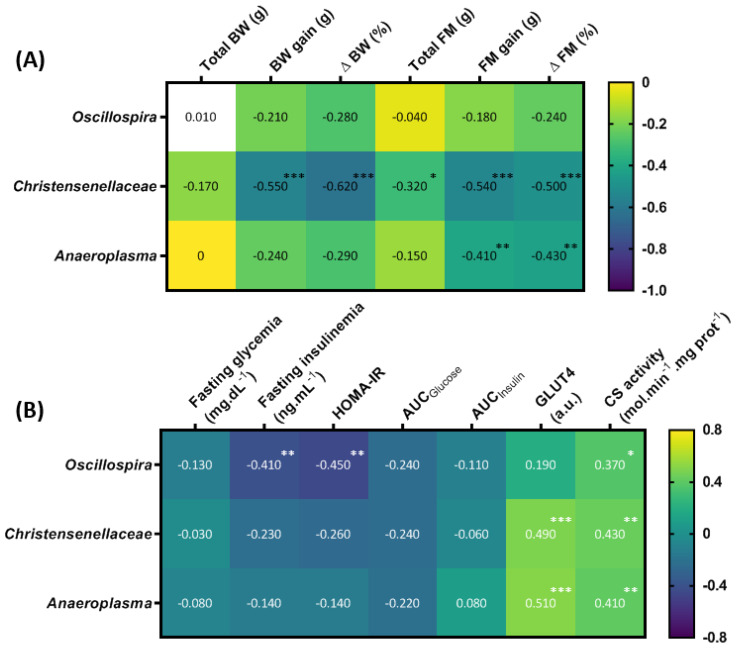
Heat map showing the association of the abundance of the indicated microbiota components with body composition (**A**), and glucose homeostasis parameters (**B**). * *p* < 0.05, ** *p* < 0.005, *** *p* < 0.005. BW: body weight; FM: fat mass; Δ: delta; AUC: area under the curve; CS: citrate synthase.

At the phylum level, Firmicutes and Bacteroidetes were the most abundant in all groups (their combined mean abundance represented 88% of all microbiota). The Tenericutes phylum was more abundant in the HIIT groups (*p* < 0.0005), especially in the HIIT + T63 group (Figure 8A). No other global phylum difference was found. At the family level, *Anaeroplasmaceae* was more abundant in the HIIT groups and particularly in the HIIT + T63 group (Figure 8B). Interestingly, the ANCOM statistical analysis highlighted significant group differences. Specifically, the relative abundance of *Anaeroplasma* and *Christensenellaceae* was higher in the HIIT than in the HFD and T63 groups, and in the HIIT + T63 group compared with HIIT alone (*p* < 0.05) (Figure 8C,D). Finally, *Oscillospira* relative abundance was significantly higher in the HIIT + T63 group than in the others (*p* < 0.0005) (Figure 8E). The relative abundance of specific microbiota components was correlated with the body composition and glucose homeostasis parameters. BW and FM gains and ΔBW and ΔFM were negatively correlated with *Christensenellaceae* relative abundance. Only FM gain and ΔFM were negatively associated with *Anaeroplasma.* ΔBW tended to be negatively correlated with *Anaeroplasma* (*p* = 0.06) and *Oscillospira* (*p* = 0.07) relative abundance (Figure 9A). Moreover, fasting insulinemia and HOMA-IR were negatively associated with *Oscillospira* relative abundance (Figure 9B). Conversely, citrate synthase activity was positively associated with the abundance of all three bacterial groups, and GLUT4 protein expression with *Christensenellaceae* and *Anaeroplasma* abundance.

## 4. Discussion

The aim of this study was to determine whether HIIT, combined with T63 supplementation or not, alters body composition and glycemic profile in a rat model of pre-obesity, specifically by modulating intestinal mucosa-associated microbiota. In our experimental conditions, the HIIT + T63 combination significantly limited BW gain, without any energy intake modulation, and improved glycemic control. BW variation was correlated with the α-diversity of the colon mucosa microbiota that was more important in the HIIT + T63 group. Moreover, the relative abundance of *Anaeroplasmaceae*, *Christensenellaceae* and *Oscillospira* was higher in the HIIT + T63 group.

The etiologies of obesity and prediabetes are very complex, due of the multiplicity of genetic and environmental determinants. However, dietary habits, sedentary time and physical inactivity play a major role in their increasing frequency. In this study, we chose a model of obesity induced by a Western-style diet rather than a genetic model, to better mimic our current lifestyle. Indeed, the Western diet, defined by high dietary intake of saturated fats and sucrose and low intake of fibers, contributes to the increased occurrence of metabolic diseases, such as diabetes and obesity [33].

Diet intervention and/or physical activity programs are necessary to fight against obesity and (pre)diabetes [34,35]. As nutritional strategy, we supplemented rats with T63, a polyphenol-rich natural supplement based on five plant extracts designed to reduce T2D risk factors. Concerning physical activity, a growing body of evidence in humans and rodents shows that HIIT is a more interesting strategy than Moderate-Intensity Continuous Training (MICT) for total and (intra-) abdominal FM reduction [36,37,38] and glucose control improvement [14,39]. In the study by Maillard et al. [38], Zucker rats performed the same HIIT protocol (6 × (3 min at 10 m·min^−1^/4 min at 18 m·min^−1^)), but only for 10 weeks, with significant body composition changes. In the present study, we added two additional weeks of training for two reasons. First, First, De Araujo et al. [40] defined short-term and long-term HIIT programs in function of their duration (<6 weeks and ≥12 weeks, respectively). Furthermore, nutritional supplementation-induced gut microbiota modulation is observed after at least 2 weeks of treatment and generally lasts less than 24 weeks [41,42,43,44]. Therefore, 12 weeks seemed a good compromise to obtain significant body composition and gut microbiota alterations, and was recently chosen also by Plissonneau et al. [45] with the same HIIT protocol.

The HIIT program alone limited BW gain up to week 24 (i.e. week 7 of the intervention period), suggesting that HIIT was effective, at least at the beginning of the intervention. After this period, it could no longer compensate the adverse effect of HFD, but was still efficient when associated with T63. Moreover, T63 supplementation and HIIT program alone led reduced BW gain by 9 and 8 g, respectively, compared with HFD, which was not negligible although not significant. Importantly, in the HIIT + T63 group, BW gain was reduced by 27 g (and not 17 g, obtained by adding the weight restrictions of the T63 and HIIT groups), suggesting a synergistic effect. Furthermore, the BW gain in the HIIT + T63 group and CTRL group (normal diet) were similar, showing that the combined effects of the two interventions completely abolished the BW gain excess induced by HFD. Concerning FM, HIIT with/without T63 supplementation significantly limited adipose tissue deposits. Interestingly, FM gain was comparable in the HIIT + T63 and CTRL groups. All these results are consistent with other studies demonstrating body composition changes with HIIT programs [38,45]. However, unlike Chavanelle et al. (2021) [17] who reported that 16-week T63 supplementation (2.7% incorporated in the food) prevents HFD-induced excessive BW and FM gain in mice, we did not observe any significant body composition effect of T63 alone. This discrepancy could be partly explained by the T63 percentage (2%) in food and the shorter period of supplementation (12 weeks) in our study. Moreover, T63 supplementation was not used to prevent, but to reverse HFD-induced obesity. Thus, our findings indicated that the HIIT and T63 combination is more effective on BW and FM gain than HIIT or T63 alone. Several studies on polyphenol supplementation and exercise reported similar results. In 2009, Maki et al. [46] showed that tea catechins combined with a physical activity program result in loss of total and abdominal FM, but not exercise alone. More recently, Ghasemi and Nayebifar [47] showed that green tea catechins combined with HIIT significantly reduce BW and FM. Considering T63 composition, we could hypothesize that polyphenols (including lutolein, oleuropein, chlorogenic acid or apigenin), in combination with exercise regulate lipolysis, ameliorate mitochondrial functions, and increase fat oxidation [48,49,50,51], leading to a significant effect on body composition. 

Chavanelle et al. (2021) [17] found that 16-week T63 supplementation prevents HFD-induced detrimental effects on glucose homeostasis in mice, with improvement of fasting insulinemia and insulin responses to the OGTT challenge. In humans, preclinical experiments showed that glucose and insulin responses during OGTT are improved after T63 supplementation for 4 weeks. HIIT effect on glycemic control are still unclear. After a 10-week HIIT program (5 × (2 min at 80–90%VO_2_max/1 min 30–35%VO_2_max)), Shanaki et al. [39] observed lower fasting glycemia, fasting insulinemia and HOMA-IR in Wistar rats with HFD-induced obesity and diabetes. Conversely, Khalafi et al. [52] did not detect any change in fasting insulinemia. Here, we did not observe any effect of HIIT alone on fasting glucose and insulin concentration and insulin responses during OGTT, but the HIIT + T63 combination significantly improved the glycemic responses during OGTT. On their own, HIIT and T63 tended to improve the glycemic responses, but the glucose AUC did not show any significant difference. Skeletal muscle is the main tissue involved in postprandial glucose uptake regulation [53]. Thus, skeletal muscle adaptations to training might partly explain HIIT positive effects on glycemic control. In humans and in rodents, long-term physical training increases GLUT4 protein expression and favors GLUT4 translocation and glucose transport in skeletal muscle [54]. Physical training also stimulates the mitochondrial content and the pool of energy metabolism enzymes, such as citrate synthase, leading to higher carbohydrate use during high-intensity exercise [53]. These adaptations are involved in whole-body glucose homeostasis. In our study, GLUT4 content in the soleus muscle tended (not significant) to be higher and OXPHOS complex protein content was significantly increased in the HIIT groups. Moreover, citrate synthase activity was significantly increased in the HIIT + T63 group. These results support the hypothesis that greater skeletal muscle oxidative capacity could partly explain the improvement of glucose tolerance during the OGTT in the training groups, especially in the HIIT + T63 group. The larger effect in the HIIT + T63 vs. HIIT group confirmed T63 impact on glucose metabolism. Several recent studies indicate that combining plant-based supplements with HIIT may enhance energy metabolism [16]. Moreover, several individual compounds included in the T63 supplement have a beneficial effect on skeletal muscle glucose regulation. For example, recent studies showed that oleuropein improves glucose uptake [55], promotes GLUT4 translocation [56], and attenuates hyperglycemia and glucose tolerance impairment in rodents [57]. 

Pro-/anti-oxidant balance modulation might promote body composition changes [58] and glucose metabolism regulation [59]. Regular physical activity reduces oxLDL and increase SOD and GP_X_ plasmatic activities [60]. Polyphenols also have anti-oxidant properties [61,62]. T63 high polyphenol content (including chlorogenic and oleanolic acids) might promote antioxidant effects [59,63]. Accordingly, in our study, oxLDL was significantly reduced in the HIIT, T63 and HIIT + T63 groups compared with HFD. Moreover, SOD activities in plasma were higher when HIIT and T63 were combined. Overall, these results suggest that HIIT and T63 supplementation could improve glucose homeostasis also by limiting plasma oxidative stress.

Finally, as metabolic diseases (like obesity and diabetes), physical activity, diet interventions and other circulating factors as antioxidants, cytokines, IGF-I, IGFBP3 are associated with changes in microbiota composition and structure [64], we evaluated the impact of HIIT and/or T63 supplementation on the microbiota associated with the intestinal mucosa. The HIIT + T63 combination increased α-diversity and modulated β-diversity of the mucosa-associated microbiota. Our analysis suggests that T63 main effect was on microbiota α- and β-diversity and that HIIT also influenced β-diversity, particularly when associated with T63. As T63 is a blend of five plant extracts, it is difficult to compare our results with literature data on specific compounds. Nevertheless, some studies demonstrated that phenolic compounds and fibers alter gut microbiota, resulting in greater abundance of beneficial bacteria [65]. On the other hand, the real impact of regular physical training on gut microbiota modulation is less clear-cut, and in rodents, conclusions are different depending on the exercise mode (voluntary/forced exercise) and nutritional status [66].

The analysis of specific mucosa-associated microbiota features highlighted several differences in the four groups. First, *Anaeroplasma* abundance was significantly increased in the two HIIT groups, especially in the HIIT + T63 group, and was correlated with FM gain (expressed as g and %), GLUT4 protein content, and soleus citrate synthase activity. *Anaeroplasma* belong to the Tenericutes phylum the abundance of which also was increased in the HIIT groups. Few studies reported Tenericutes phylum changes after physical training, but Allen et al. showed that a 6-week treadmill training increases Tenericutes, like in the present study. Recently, Yuan et al. [67] demonstrated that polysaccharide-rich extracts could reverse the gut microbiota dysbiosis observed in diabetic mice by increasing the abundance of *Anaeroplasma*. *Christensenellaceae* abundance also was increased in our HIIT groups, particularly in the HIIT + T63 group, compared with the HFD and T63 groups. Its abundance was negatively correlated with BW and FM gains, and positively associated with GLUT4 protein content and citrate synthase activity. Similarly, *Christensenellaceae* abundance was increased after 6 weeks of training and energy restriction in adolescents with obesity [68]. Interestingly, this genus is associated with healthy BMI values and reduces diet-induced weight gain in mice [69] and in humans [70]. Recently, elevated gut microbiome abundance of *Christensenellaceae* was also associated with reduced visceral adipose tissue and healthier metabolic profile in Italian older adults [71]. Interestingly, this bacterium is depleted in inflammatory bowel diseases [72], further confirming its beneficial effect. Finally, *Oscillospira* abundance was significantly increased only in the HIIT + T63 group, and was negatively correlated with fasting insulinemia and HOMA-IR and positively correlated with citrate synthase activity. Haro et al. [73] found higher *Oscillospira* abundance in people with obesity following a Mediterranean, polyphenol- and fiber-rich diet. Surprisingly, T63 supplementation alone did not influence *Oscillospira* abundance. However, different studies showed a negative correlation between *Oscillospira* and glycemic parameters [74,75], like in the present work. Moreover, several studies found that *Oscillospira* abundance in microbiota is increased by physical activity [38,66]. Specifically, our laboratory recently demonstrated that a 12-week HIIT program increases *Oscillospira* abundance that is negatively correlated with BW and FM changes [45]. Taken together, these results suggest that *Anaeroplasmaceae, Christensenellaceae* and *Oscillospira* strains could be a promising new generation of probiotics in the context of obesity and T2D.

There is inter-individual variability in the metabolic responses to physical training [76]. Different factors, such as genetic and epigenetic features, nutrition, concomitant treatments, may explain this heterogeneity [77,78]. Gut microbiota composition also may be a potential factor to explain the training response variability [79]. Similarly, the metabolic adaptations to nutritional interventions also show variability, particularly upon exposure to polyphenolic compounds [80]. Moreover, metabolic adaptations induced by nutritional interventions might be influenced by the gut microbiota composition before the supplementation/diet [44,81]. Altogether, these findings suggest that the greater results of the HIIT + T63 combination on body composition and glucose metabolism in our study might be explained by its effect on the gut microbiota composition.

## 5. Conclusions

Obesity and prediabetes are the two strongest risks factors of T2D. Therefore, it is important to identify non-drug strategies to prevent and treat obesity and glucose tolerance impairment. Our preclinical study using an animal model of obesity induced by a Western-style diet showed that physical activity combined with T63, a natural-rich polyphenol plant extract supplement, limits FM gain, and improves glycemic control by increasing skeletal muscle oxidative capacity. Changes in the intestinal mucosa-associated microbiota (particularly *Anaeroplasma*, *Christensenellaceae* and *Oscillospira*) were more important in the HIIT + T63 group. Thus, the combination of HIIT and T63 supplementation could be proposed for the management of obesity and prediabetes and also of other chronic pathologies involving gut microbiota dysbiosis.

## Figures and Tables

**Table 1:** Chemical characterization of whole TOTUM-63 powder. Reprinted with permission from ref. [17] Copyright 2021 VALBIOTIS.

Compound Type	Extract Content (g/100 g Dry Weight)
Total polyphenols	14.36
Total anthocyanins	0.81
Monocaffeoylquinic acids	1.18
Chlorogenic acid	0.85
Other monocaffeoylquinic acids	0.33
Dicaffeoylquinic acids	0.98
Cynarin	0.24
Other dicaffeoylquinic acids	0.74
Caffeic acid	0.01
Oleuropein	3.72
Oleuropein isomers	0.20
Hydroxytyrosol	0.04
Luteolin	0.01
Luteolin-7-O-glucoside	0.38
Luteolin-7-O-glucuronide	0.38
Apigenin	0.01
Apigenin-7-O-glucoside	0.01
Apigenin-7-O-glucuronide	0.25
Apigenin-6-C-glucoside-8-C-arabinoside (shaftoside)	0.06
Apigenin-6,8-C-diglucoside (vicenin 2)	0.06
Eriodictyol	<0.01
Eriodictyol-7-O-glucoside	0.11
Okanin-4-O-glucoside (marein)	0.05
Isookanin-7-O-glucoside (flavanomarein)	0.05
Maritimetin-6-O-glucoside (maritimein)	0.08
Saponins	
Chrysantellin A	0.01
Chrysantellin B	0.27
Alkaloids	
Piperine	0.004
Fibers	
Soluble fibers	13.7
Insoluble fibers	3.3

**Table 2:** Primers used for real-time PCR analyses. IL, interleukin; *Tnf*α, tumor necrosis factors-α; *Ppparg*1α, peroxisome proliferator-activated receptor coactivator-1 α; *Nrf1*, nuclear respiratory factor 1; *Gapdh*, glyceraldehyde 3-phosphate dehydrogenase.

Genes	Forward Primer 5′ → 3′	Reverse Primer 5′ → 3′
*Il1β*	ATCTCACAGCAGCATCTCGA	TAGCAGGTCGTCATCATCCC
*Il6*	CCACTGCCTTCCCTACTTCA	TTCTGACAGTGCATCATCGC
*Il10*	AGAGAACCATGGCCCAGAAA	TGAGTGTCACGTAGGCTTCT
*Tnfα*	TCATCCGTTCTCTACCCAGC	TACTTCAGCGTCTCGTGTGT
*Ppargc1α*	AATGCAGCGGTCTTAGCACT	GTGTGAGGAGGGTCATCGTT
*Nrf1*	TTCCTCAGCCTCCGTCTTCT	ACACACCTTGCACTCACACC
*Gapdh*	CATGCCATCACTGCCACTCA	GCGGCATGTCAGATCCACAA

**Table 3:** Fecal acetate, butyrate and propionate concentration (µmol·g^−1^ feces) at the end of intervention period. SCFAs, Short chain fatty acids; Total SCFAs = acetate + butyrate + propionate.

	HFD	T63	HIIT	HIIT+T63	*p*	*η^2^*
Acetate	26.1 ± 4.3	22.5 ± 5.6	26.7 ± 7.1	23.5 ± 3.1	0.24	0.11
Butyrate	2.1 ± 1.0	2.1 ± 1.4	3.1 ± 1.9	1.6 ± 0.6	0.15	0.14
Propionate	2.4 ± 0.8	2.2 ± 0.9	2.8 ± 1.7	1.9 ± 0.3	0.30	0.09
Total SCFAs	30.8 ± 5.5	26.8 ± 7.3	32.5 ± 9.9	27.1 ± 3.7	0.21	0.12

## Supplementary Materials

The following are available online at https://www.mdpi.com/article/10.3390/nu13051569/s1, Figure S1: Effects of T63 supplementation and/or HIIT on GLUT4 protein content (A), citrate synthase (CS) activity (B), and OXPHOS complex protein content (C) in gastrocnemius. * *p* < 0.05, ** *p* < 0.005, *** *p* < 0.0005, Figure S2: Effects of T63 supplementation and/or HIIT on Ppargc1α (A) and Nrf1 (B) gene expression in soleus. * *p* < 0.05, Figure S3: Effects of T63 supplementation and/or HIIT on advanced oxidation protein products (AOPP), superoxide dismutase (SOD) activity, and glutathione peroxidase (GPx) in gastrocnemius (A-B-C), epididymal adipose tissue (D-E-F) and in liver (G-H-I), Figure S4: Effects of T63 supplementation and/or HIIT on myeloperoxidase (MPO) (A), and lipopolysaccharide binding protein (LBP) (B) concentration in plasma, Figure S5: Effects of T63 supplementation and/or HIIT on inflammatory gene expression in colon (A-B-C-D), epididymal adipose tissue (E-F-G-H) and in subcutaneous adipose tissue (I-J-K).
