# Supplementary material for: The TOTUM-63 Supplement and High-Intensity Interval Training Combination Limits Weight Gain, Improves Glycemic Control, and Influences the Composition of Gut Mucosa-Associated Bacteria in Rats on a High Fat Diet
Source: Nutrients. 2021 May 7;13(5):1569. doi: 10.3390/nu13051569 (PMC8151333; doi:10.3390/nu13051569)
Supplement: Supplementary file 1 [file nutrients-13-01569-s001.zip › Supplementary-Data-1.pdf]

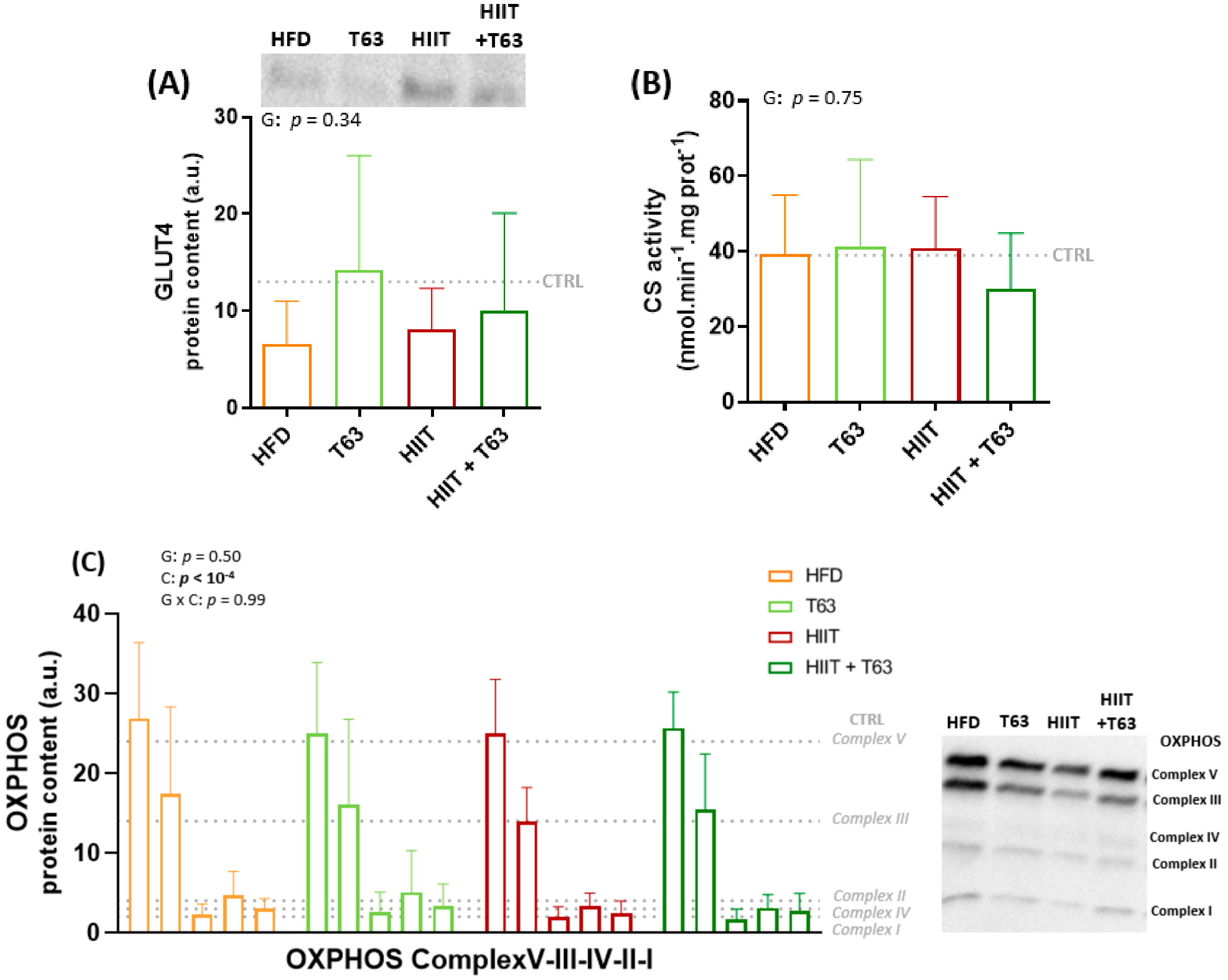

Figure S1: Effects of T63 supplementation and/or HIIT on GLUT4 protein content (A), citrate synthase (CS) activity (B), and OXPHOS complex protein content (C) in gastrocnemius. *$p < 0.05$, **$p < 0.005$, ***$p < 0.0005$.
