# Supplementary material for: The TOTUM-63 Supplement and High-Intensity Interval Training Combination Limits Weight Gain, Improves Glycemic Control, and Influences the Composition of Gut Mucosa-Associated Bacteria in Rats on a High Fat Diet
Source: Nutrients. 2021 May 7;13(5):1569. doi: 10.3390/nu13051569 (PMC8151333; doi:10.3390/nu13051569)
Supplement: Supplementary file 1 [file nutrients-13-01569-s001.zip › Supplementary-Data-3.pdf]

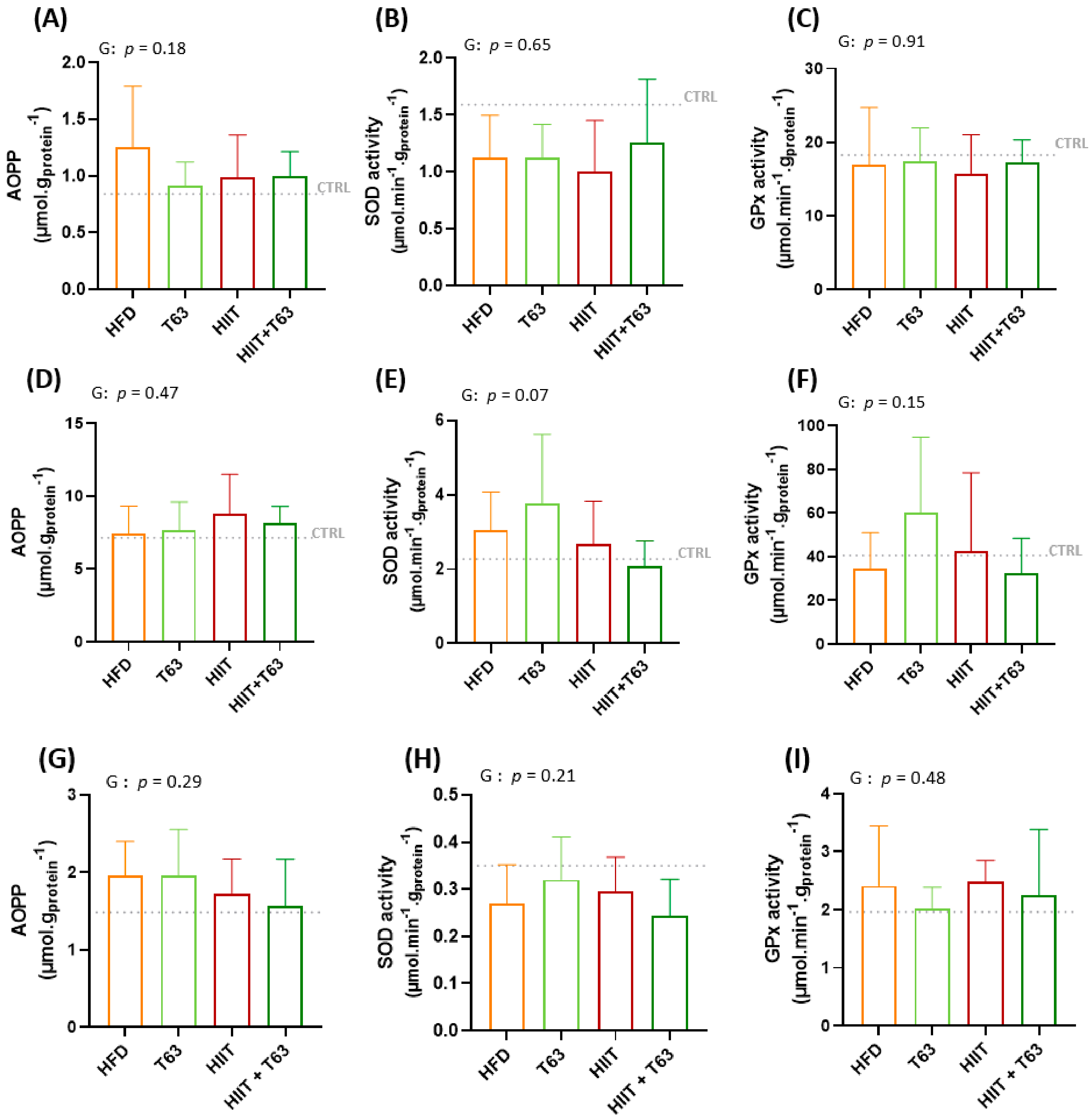

Figure S3: Effects of T63 supplementation and/or HIIT on advanced oxidation protein products (AOPP), superoxide dismutase (SOD) activity, and glutathione peroxidase (GPx) in gastrocnemius (A-B-C), epididymal adipose tissue (D-E-F) and in liver (G-H-I).
