# Supplementary material for: The TOTUM-63 Supplement and High-Intensity Interval Training Combination Limits Weight Gain, Improves Glycemic Control, and Influences the Composition of Gut Mucosa-Associated Bacteria in Rats on a High Fat Diet
Source: Nutrients. 2021 May 7;13(5):1569. doi: 10.3390/nu13051569 (PMC8151333; doi:10.3390/nu13051569)
Supplement: Supplementary file 1 [file nutrients-13-01569-s001.zip › Supplementary-Data-4.pdf]

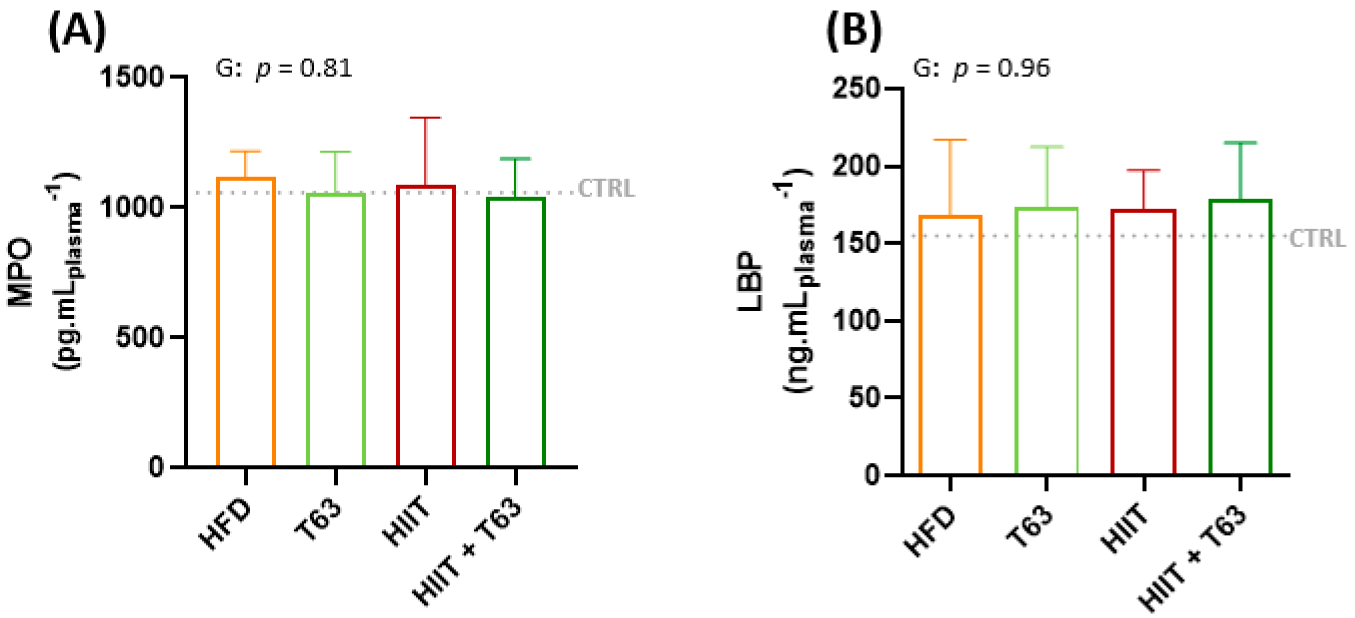

Figure S4: Effects of T63 supplementation and/or HIIT on myeloperoxidase (MPO) (A), and lipopolysaccharide binding protein (LBP) (B) concentration in plasma.
