# Supplementary material for: The TOTUM-63 Supplement and High-Intensity Interval Training Combination Limits Weight Gain, Improves Glycemic Control, and Influences the Composition of Gut Mucosa-Associated Bacteria in Rats on a High Fat Diet
Source: Nutrients. 2021 May 7;13(5):1569. doi: 10.3390/nu13051569 (PMC8151333; doi:10.3390/nu13051569)
Supplement: Supplementary file 1 [file nutrients-13-01569-s001.zip › Supplementary-Data-5.pdf]

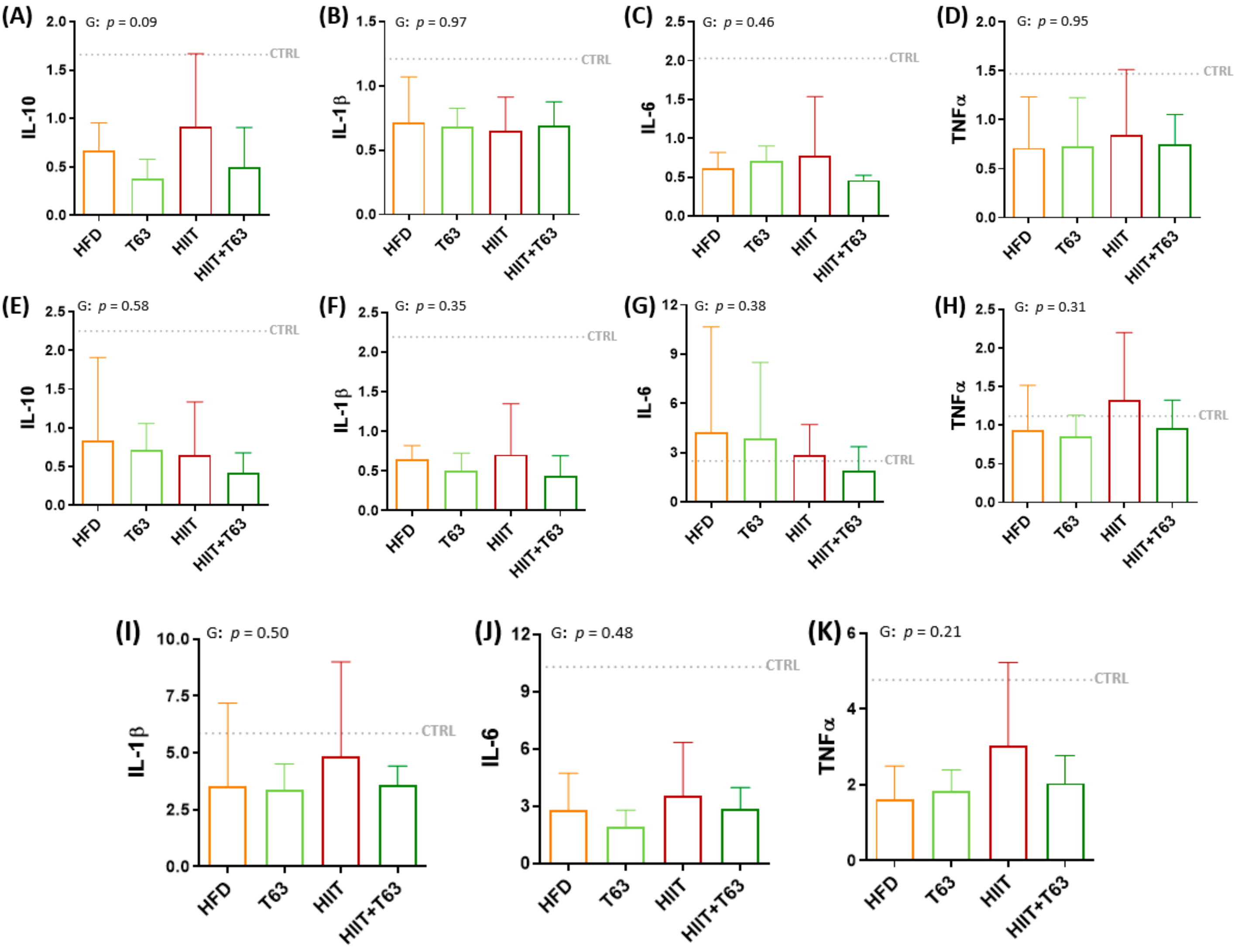

Figure S5: Effects of T63 supplementation and/or HIIT on inflammatory gene expression in colon (A-B-C-D), epididymal adipose tissue (E-F-G-H) and in subcutaneous adipose tissue (I-J-K).
